# Trends in utilization of lipid- and blood pressure-lowering agents and goal attainment among the U.S. diabetic population, 1999-2008

**DOI:** 10.1186/1475-2840-10-31

**Published:** 2011-04-17

**Authors:** Andreas Kuznik, Jack Mardekian

**Affiliations:** 1 Pfizer Inc., New York, New York, USA

## Abstract

**Background:**

For patients with diabetes, clinical practice guidelines recommend treating to a low-density lipoprotein cholesterol (LDL-C) goal of <2.59 mmol/L (100 mg/dL) and a blood pressure (BP) target of <130/80 mmHg. This analysis assessed recent trends in the utilization of lipid-lowering and BP-lowering agents, as well as LDL-C and BP goal attainment, in the U.S. adult diabetic population.

**Methods:**

9,167 men and nonpregnant women aged ≥20 years were identified from the fasting subsample of the 1999-2008 National Health and Nutritional Examination Survey. Diabetes was identified in 1,214 participants by self report, self-reported use of insulin or oral medications for diabetes, or fasting glucose ≥6.99 mmol/L (126 mg/dL).

**Results:**

The prevalence of diagnosed or undiagnosed diabetes increased significantly over the past decade, from 7.4% in 1999-2000 to 11.9% in 2007-2008 (*P *= 0.0007). During this period, the use of lipid-lowering agents by participants with diabetes increased from 19.5% to 42.2% (*P *< 0.0001), and the proportion at LDL-C goal increased from 29.7% to 54.4% (*P *< 0.0001). Although there was a significant increase in antihypertensive medication use (from 35.4% to 58.9%; *P *< 0.0001), there was no significant change in the proportion of participants at BP goal (from 47.6% to 55.1%; *P *= 0.1333) or prevalence of hypertension (from 66.6% to 74.2%; *P *= 0.3724).

**Conclusions:**

The proportion of diabetic individuals taking lipid- and BP-lowering agents has increased significantly in recent years. However, while there has been a significant improvement in LDL-C goal attainment, nearly one-half of all U.S. adults with diabetes are not at recommended LDL-C or BP treatment goals.

## Background

An estimated 18.8 million Americans have a diagnosis of diabetes, and a further 7 million have undiagnosed diabetes [1]. A recent analysis including diagnosed diabetes, undiagnosed diabetes, and pre-diabetes indicates that >40% of the U.S. adult population has some form of hyperglycemia [2]. The economic burden associated with diabetes is substantial: the total cost attributable to recognized diabetes in the U.S. in 2007 was estimated at $174 billion [3].

Cardiovascular (CV) complications are the major contributors to morbidity and mortality in patients with diabetes [4]. The risk of mortality from cardiovascular disease (CVD) is increased by up to 5-fold in patients with diabetes [5-7]. Although debated within the literature, the National Cholesterol Education Program Adult Treatment Panel III has classified diabetes as a coronary heart disease (CHD) risk equivalent [8,9]. Dyslipidemia and hypertension are independent predictors of future CV events, and clinical interventions that target these risk factors have been shown to reduce CV outcomes in patients with diabetes [10-16]. As such, aggressive control of modifiable CV risk factors is particularly important in this high-risk population.

Current national treatment guidelines for patients with diabetes [4] advocate a low-density lipoprotein cholesterol (LDL-C) goal of <2.59 mmol/L (100 mg/dL), with an optional goal of <1.81 mmol/L (70 mg/dL) in those with overt CVD, and a blood pressure (BP) goal of <130/80 mmHg. With respect to lipid-lowering therapy, treatment recommendations include the use of statins in addition to lifestyle modification to improve lipid profiles. For diabetic patients with overt CVD, or those without CVD but >40 years of age with ≥1 other CVD risk factor, statin therapy is recommended irrespective of baseline lipid levels.

Using National Health and Nutritional Examination Survey (NHANES) data covering the period 1999-2008, this analysis of the U.S. adult diabetic population assessed recent trends in the utilization of lipid-lowering and antihypertensive agents, as well as LDL-C and BP goal attainment rates.

## Research design and methods

### Study design

NHANES is conducted by the National Center of Health Statistics, Centers for Disease Control and Prevention, as a cross-sectional, stratified, multistage probability sample survey of the U.S. civilian, noninstitutionalized population [17,18]. NHANES data are derived from direct interviews regarding medical history, medication use, and diet, as well as clinical examinations (including BP measurements) and laboratory tests (including lipid and glucose blood biochemistries). From 1999, NHANES became a continuous survey, and data are released in 2-year increments. This analysis used data from the 5 most recent study cycles: 1999-2000, 2001-2002, 2003-2004, 2005-2006, and 2007-2008. NHANES 1999-2008 received approval from the National Center for Health Statistics research ethics review board, and written informed consent was obtained from all NHANES participants [17].

### Sample population

A total of 9,167 men and nonpregnant women ≥20 years of age with valid data on their diabetic status, in addition to complete lipid and BP data, were identified from the fasting subsample (n = 16,675) of the 1999-2008 NHANES population. NHANES participants are randomly selected for inclusion in the fasting subsample and instructed to fast for 8 to <24 hours prior to blood specimens being taken for laboratory testing [18]. Presence of diagnosed or undiagnosed diabetes in a subset of 1,214 participants was identified by self report of diabetes, self-reported use of insulin or oral medications for diabetes, or a fasting plasma glucose ≥6.99 mmol/L (126 mg/dL).

### Data collection and laboratory measurements

All drug utilization and disease history was self-reported. BP measurements in NHANES were performed 3-4 times manually with a mercury sphygmomanometer according to a standard protocol [18]; the first reading was excluded and remaining readings used to compute average BP. Hypertension was defined as an average BP >130 mmHg systolic or >80 mmHg diastolic, or self-reported use of antihypertensive agents.

Methods for determining blood lipid and glucose levels in NHANES have been described [18]. Briefly, total cholesterol was measured enzymatically on the basis of hydrogen peroxide generation. In 1999-2002, high-density lipoprotein cholesterol (HDL-C) was measured using two methods, heparin-manganese precipitation and direct immunoassay, depending on participant age and specimen volume. From 2003, all HDL-C measurements used the direct immunoassay method. Triglycerides were measured after hydrolysis to glycerol. LDL-C levels were calculated from measurements of total cholesterol, triglycerides (≤4.52 mmol/L [400 mg/dL]), and HDL-C according to the Friedewald calculation. Non-HDL-C was calculated as total cholesterol minus HDL-C. Plasma glucose was measured using a modified hexokinase enzymatic method.

### Definition of treatment goals

Participants were classified as meeting current LDL-C and BP treatment goals for patients with diabetes [4] if their LDL-C level was <2.59 mmol/L (100 mg/dL) or their BP was ≤130/80 mmHg. In a subset of participants with a history of CVD, attainment of the optional LDL-C goal of <1.81 mmol/L (70 mg/dL) was also examined. In addition, attainment of the secondary lipid goals of an HDL-C level >1.04 mmol/L (40 mg/dL) in men or >1.30 mmol/L (50 mg/dL) in women, and a non-HDL-C level <3.37 mmol/L (130 mg/dL), were also assessed.

### Statistical methods

Statistical analyses were performed using SAS software version 9.2 (SAS Institute Inc., Cary, North Carolina). Estimated population prevalences with *P*-values were calculated using the SURVEYFREQ, SURVEYLOGISTIC, and SURVEYREG procedures in SAS, and were standardized to the July 2008 U.S. census population ≥20 years of age (n = 221,419,638). NHANES fasting sample weights were used and estimates obtained are representative of the U.S. population. Statistical tests assessing linear trends over the five 2-year survey cycles were 2-sided and a *P*-value < 0.05 was considered statistically significant.

## Results

### Prevalence of diabetes

**Figure 1:**
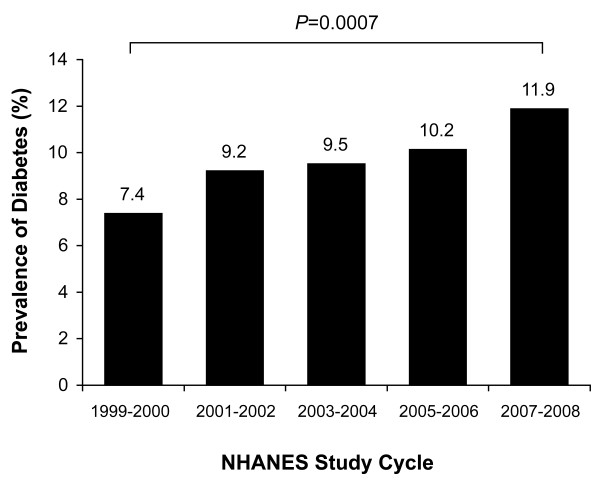
**Prevalence of diabetes in NHANES, 1999-2008**. Diabetes was identified by self report, self-reported use of insulin or oral medications for diabetes, or fasting glucose ≥6.99 mmol/L (126 mg/dL).

The prevalence of diagnosed or undiagnosed diabetes in NHANES during the period from 1999 to 2008 is presented in Figure 1 by 2-year survey cycle. Diabetes prevalence increased significantly over the 5 NHANES study cycles, from 7.4% in 1999-2000 to 11.9% in 2007-2008 (*P *= 0.0007).

### Characteristics of participants with diabetes

The demographic and clinical characteristics of 1999-2008 NHANES participants with diabetes are shown in Table 1. The majority of diabetic participants had a diagnosis of diabetes (75.1% in 2007-2008). Compared with the 1999-2000 diabetic cohort, participants with diabetes in the 2007-2008 survey were older (60.0 vs 55.4 years; *P *= 0.0146), had lower systolic (129.7 vs 130.8 mmHg; *P *= 0.0198) and diastolic (68.7 vs 71.5 mmHg; *P *< 0.0001) BP levels, lower total cholesterol (4.7 vs 5.2 mmol/L [180.6 vs 201.7 mg/dL]; *P *= 0.0001), LDL-C (2.6 vs 3.2 mmol/L [102.3 vs 121.9 mg/dL]; *P *< 0.0001), and triglyceride (1.6 vs 2.1 mmol/L [145.8 vs 186.7 mg/dL]; *P *= 0.0285) levels, and a higher HDL-C level (1.3 vs 1.1 mmol/L [49.2 vs 42.6 mg/dL]; *P *< 0.0001). Although a statistically significant increase in body mass index was observed in the 2007-2008 versus 1999-2000 cohort (32.0 vs 31.9 kg/m^2^; *P *= 0.0072), such a small numerical increase is likely to be of limited clinical significance even at a population level. Participants in the most recent survey cycle were also more likely to be taking antidiabetic medications (66.2% vs 40.3%; *P *< 0.0001), as well as medications for hypercholesterolemia and hypertension (discussed below). CVD burden was greater in the 2007-2008 cohort versus the 1999-2000 cohort, most notably for stroke (9.3% vs 3.5%; *P *= 0.0104).

**Table 1:** Characteristics of NHANES participants with diabetes*, 1999-2008

	1999-2000 (n = 149)		2001-2002 (n = 220)		2003-2004 (n = 209)		2005-2006 (n = 240)		2007-2008 (n = 396)		
						
Characteristic	n	Value	n	Value	n	Value	n	Value	n	Value	*P*†
Diabetes diagnosed (%)	98	70.3 (5.0)	144	69.2 (4.1)	158	72.0 (3.6)	182	73.0 (3.9)	303	75.1 (2.1)	0.2203
Age at screening (years)	149	55.4 (1.7)	220	57.0 (1.2)	209	59.1 (1.8)	240	59.2 (1.3)	396	60.0 (0.8)	0.0146
Male (%)	70	52.7 (4.3)	124	58.4 (4.2)	112	56.3 (3.6)	120	45.0 (4.5)	204	52.2 (3.2)	0.2156
Race/ethnicity (%)											0.9490
Non-Hispanic white	51	66.5 (6.8)	100	65.3 (5.3)	93	66.9 (7.5)	104	66.0 (5.6)	179	66.7 (5.6)	
Non-Hispanic black	30	11.9 (2.6)	46	13.9 (2.2)	40	13.0 (3.6)	75	16.7 (3.0)	91	16.0 (3.9)	
Mexican American	53	6.4 (1.9)	56	6.5 (1.2)	61	8.4 (3.8)	52	9.9 (2.0)	70	8.4 (2.4)	
Other	15	15.2 (7.4)	18	14.3 (5.3)	15	11.6 (3.5)	9	7.4 (2.8)	56	8.9 (2.6)	
Body mass index (kg/m^2^)	148	31.9 (1.1)	208	31.9 (1.1)	203	31.0 (0.7)	234	33.4 (0.5)	383	32.0 (0.5)	0.0072
Blood pressure (mmHg)											
Systolic	149	130.8 (1.3)	220	131.8 (1.5)	209	129.5 (2.0)	240	130.5 (1.7)	396	129.7 (1.4)	0.0198
Diastolic	149	71.5 (2.1)	220	71.7 (1.4)	209	69.1 (1.4)	240	68.1 (1.2)	396	68.7 (0.9)	<0.0001
Total cholesterol (mg/dL)	149	201.7 (4.1)	220	195.6 (2.6)	209	198.3 (3.2)	240	191.4 (3.5)	396	180.6 (2.6)	0.0001
LDL cholesterol (mg/dL)	149	121.9 (3.1)	220	116.6 (2.1)	209	114.3 (2.9)	240	107.4 (2.6)	396	102.3 (2.3)	<0.0001
HDL cholesterol (mg/dL)	149	42.6 (1.3)	220	48.0 (1.6)	209	49.8 (1.4)	240	52.4 (1.6)	396	49.2 (0.8)	<0.0001
Triglycerides (mg/dL)	149	186.7 (8.3)	220	155.5 (4.6)	209	170.6 (9.2)	240	157.7 (5.7)	396	145.8 (2.9)	0.0285
Medication use (%)‡											
Antidiabetics§	72	40.3 (7.6)	113	52.2 (4.0)	125	53.4 (4.0)	166	65.9 (4.0)	268	66.2 (2.8)	<0.0001
Antihyperlipidemics║	31	19.5 (3.9)	54	23.9 (3.8)	81	42.4 (3.5)	97	40.6 (4.3)	163	42.2 (3.6)	<0.0001
Antihypertensives¶	63	35.4 (5.4)	114	46.2 (4.3)	116	49.6 (3.5)	145	59.7 (2.7)	236	58.9 (3.1)	<0.0001
Cardiovascular disease history (%)‡											
Coronary heart disease#	26	15.4 (4.7)	38	12.9 (2.4)	37	18.6 (4.9)	42	16.0 (1.9)	79	20.9 (2.3)	0.1603
Congestive heart failure	9	5.8 (2.8)	17	5.3 (1.4)	15	6.4 (2.5)	24	9.4 (1.7)	36	9.0 (1.8)	0.1314
Stroke	4	3.5 (2.3)	11	3.9 (1.4)	18	8.3 (2.1)	31	11.7 (3.5)	39	9.3 (2.2)	0.0104
Hypertension (%)**	109	66.6 (7.2)	176	76.4 (2.5)	155	69.4 (4.3)	188	75.5 (2.9)	307	74.2 (2.6)	0.3724

Values are weighted percent (standard error) or mean (standard error). Estimates were standardized to the July 2008 U.S. census population ≥20 years of age.

To convert total cholesterol, LDL cholesterol, HDL cholesterol from mg/dL to mmol/L, multiply by 0.0259; to convert triglycerides from mg/dL to mmol/L, multiply by 0.0113.

*Diabetes was identified by self report, self-reported use of insulin or oral medications for diabetes, or fasting glucose ≥6.99 mmol/L (126 mg/dL). †*P *values are for 1999-2000 versus 2007-2008; *P *value for race/ethnicity compares the distribution of race/ethnicity categories. ‡All drug utilization and disease history was self-reported. §Any antidiabetic agents including insulin and oral medications for diabetes. ║Any lipid-lowering agents including statins, fibric acid derivatives, bile acid sequestrants, cholesterol absorption inhibitors, and other antihyperlipidemic agents. ¶Any antihypertensive agents including β-blockers, calcium channel blockers, diuretics, angiotensin-converting enzyme inhibitors, angiotensin receptor blockers, and other BP-lowering agents. #Coronary heart disease was identified by self report of heart disease, angina, or myocardial infarction. **Hypertension was defined as an average BP >130 mmHg systolic or >80 mmHg diastolic, or self-reported use of antihypertensive agents.

### Lipid control in participants with diabetes

**Figure 2:**
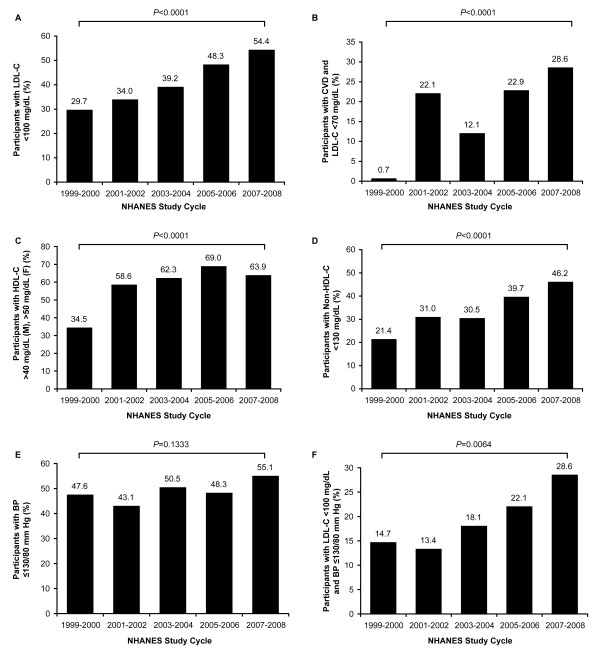
**Lipid and BP Goal Attainment in NHANES participants with diabetes, 1999-2008**. A: Proportion of participants at LDL-C goal <2.59 mmol/L (100 mg/dL). B: Proportion of participants with CVD history at LDL-C goal <1.81 mmol/L (70 mg/dL). CVD was identified by self report of CHD, congestive heart failure, or stroke; CHD was identified by self report of heart disease, angina, or myocardial infarction. C: Proportion of participants at HDL-C goal >1.04 mmol/L (40 mg/dL, in males, "M") or >1.30 mmol/L (50 mg/dL, in females, "F"). D: Proportion of participants at non-HDL-C goal <3.37 mmol/L (130 mg/dL). E: Proportion of participants at BP goal ≤130/80 mmHg. F: Dual LDL-C (<2.59 mmol/L [100 mg/dL]) and BP (≤130/80 mmHg) goal attainment. To convert total cholesterol, LDL-C, HDL-C, and non-HDL-C from mg/dL to mmol/L, multiply by 0.0259.

During the survey period examined, there was a significant increase in the self-reported use of lipid-lowering agents by participants with diabetes, from 19.5% in 1999-2000 to 42.2% in 2007-2008 (*P *< 0.0001; Table 1). Over the same time frame, the proportion of participants achieving the LDL-C goal of <2.59 mmol/L (100 mg/dL) also increased significantly, from 29.7% to 54.4% (*P *< 0.0001; Figure 2A). In the subset of diabetic participants with a history of CVD, attainment of the optional LDL-C goal of <1.81 mmol/L (70 mg/dL) increased from 0.7% in 1999-2000 to 28.6% in 2007-2008 (*P *< 0.0001; Figure 2B). An analysis of the proportion of participants with diabetes achieving the secondary lipid goal of an HDL-C level >1.04 mmol/L (40 mg/dL) in men or >1.30 mmol/L (50 mg/dL) in women revealed an increase from 34.5% to 63.9% over the study period (*P *< 0.0001; Figure 2C). Attainment of a non-HDL-C goal of <3.37 mmol/L (130 mg/dL) increased from 21.4% in 1999-2000 to 46.2% in 2007-2008 (*P *< 0.0001; Figure 2D).

### BP control in participants with diabetes

The self-reported use of antihypertensive agents by participants with diabetes increased significantly over the 5 study cycles, from 35.4% in 1999-2000 to 58.9% in 2007-2008 (*P *< 0.0001; Table 1). However, despite the increased utilization of BP-lowering medications over time, there was no significant change in the proportion of participants achieving the BP goal of ≤130/80 mmHg (47.6% in 1999-2000; 55.1% in 2007-2008; *P *= 0.1333; Figure 2E) or the prevalence of hypertension (which increased from 66.6% to 74.2%; *P *= 0.3724; Table 1).

### Dual LDL-C and BP goal attainment in participants with diabetes

The proportion of participants with diabetes who simultaneously achieved both an LDL-C <2.59 mmol/L (100 mg/dL) and a BP ≤130/80 mmHg increased significantly over the survey period, from 14.7% in 1999-2000 to 28.6% in 2007-2008 (*P *= 0.0064; Figure 2F).

## Discussion

This analysis of 1999-2008 NHANES data has revealed that the prevalence of diabetes among U.S. adults increased significantly over the past decade. In 2007-2008, 11.9% of participants ≥20 years of age had either diagnosed or undiagnosed diabetes, representing an estimated 26.4 million Americans. Concomitant with the rise in diabetes prevalence was an increase in the utilization of lipid-lowering and antihypertensive medications in these individuals. Although there was a significant improvement in LDL-C goal attainment rates over the 10-year period, the proportion of participants at BP goal remained relatively stable, as did the prevalence of hypertension. Hence, despite the observed increase in the pharmacological treatment of CV risk factors within these high-risk individuals, nearly one-half of the U.S. diabetic population is currently not at recommended LDL-C or BP therapeutic goals. Moreover, only ~1 in 4 people with diabetes are achieving both the LDL-C and BP treatment targets simultaneously, with a similar proportion of diabetic participants considered to be at very high risk of future CV events (those with a history of CVD) reaching the more stringent LDL-C goal of <1.81 mmol/L (70 mg/dL).

These latest estimates of diabetes prevalence and CV risk factor control in the U.S. diabetic population extend upon previous reports derived from NHANES data to 2006. Data from NHANES I (1971 to 1975), NHANES II (1976 to 1980), NHANES III (1988 to 1994), and NHANES 1999 to 2004 showed a linearly decreasing trend of participants who reported not having been diagnosed with diabetes [19]. For the period 2005-2006, one analysis estimated that 12.9% of U.S. adults had diabetes, which included diagnosed diabetes and undiagnosed diabetes based on either an oral glucose tolerance test or fasting plasma glucose [2]. The prevalence of diabetes based on diagnosis or fasting plasma glucose only, which is more in line with the criteria for diabetes used in this current analysis, was ~10%. Another study, which included only individuals with diagnosed diabetes, found a prevalence rate for 2003-2006 of 7.8% [20]. Recent analyses of NHANES data suggest that, based on fasting plasma glucose levels, an additional 2%-3% of the U.S. population have undiagnosed diabetes [2,3,21]. Our extended analysis has shown that diabetes prevalence has increased by nearly 2% in the past 4 years, from 10.2% in 2005-2006 to 11.9% in 2007-2008, an increase of ~4.3 million people. Previous increases in diabetes prevalence rates have been driven by a rise in diagnosed diabetes, with the prevalence of undiagnosed diabetes remaining stable [2,21], and may be attributable to increased awareness of the condition among healthcare professionals and the general public. However, this analysis has shown that around one-quarter of diabetes in the U.S. adult population is currently undiagnosed, indicating the potential for further improvement in the recognition of diabetes and its associated risk factors.

Few studies have examined LDL-C goal attainment in the general U.S. diabetic population [20,22], with most assessing the achievement of a total cholesterol target of <5.18 mmol/L (200 mg/dL) [22-24]. An analysis of data from NHANES 1988-1994 (NHANES III) and NHANES 1999-2002, in addition to Behavioral Risk Factor Surveillance System data from 1995 and 2002, found a substantial increase in the proportion of the adult diabetic population achieving the less-stringent LDL-C target of <3.37 mmol/L (130 mg/dL) between the baseline surveys (42.4%) and later surveys (64.2%); an LDL-C <2.59 mmol/L (100 mg/dL) was attained in 10.8% and 33.8% of participants with diabetes, respectively [22]. Estimates from more recent NHANES study cycles found that the proportion of adults with diagnosed diabetes achieving an LDL-C target of <2.59 mmol/L (100 mg/dL) increased significantly, from 36.1% in 1999-2002 to 46.5% in 2003-2006 [20]. Our results indicate that this positive trend in LDL-C goal attainment among U.S. adults with diabetes is continuing, with around half (54.4%) of this population achieving the therapeutic target in 2007-2008.

The same cannot be said of BP goal attainment. Almost without exception [24], previous analyses of national population-based data sets have reported a lack of significant improvement in BP control over time in those with diagnosed diabetes [20,22,23]. Although we observed a numerical increase (to 55.1%) in the proportion of U.S. diabetic individuals with a BP ≤130/80 mmHg, the overall trend in BP goal attainment over the 10-year period was not significant.

### Undertreatment of dyslipidemia in patients with diabetes

Despite an observed increase in the use of lipid-lowering agents by the U.S. diabetic population over the survey period, in the most recent study cycle (2007-2008) less than one-half (42.2%) of these high-risk individuals reported taking some form of lipid-modifying therapy, and just over one-half (54.4%) were achieving the LDL-C goal recommended by current national treatment guidelines for diabetes. Undertreatment of dyslipidemia in diabetes has also been reported in the real-world clinical setting: a recent evaluation of the pharmacological treatment of mixed dyslipidemia (suboptimal LDL-C, HDL-C, and/or triglyceride levels) in diabetic patients from a large U.S. managed healthcare plan found that >40% received no lipid-lowering therapy even after suboptimal lipid values were obtained [25]. Underutilization of lipid-lowering drugs in patients with diabetes is not restricted to the U.S. but is a global problem that also affects Europe [e.g. ref. [26]] and Asia [e.g. ref. [27]]. An analysis of the German DUTY diabetes registry found that just 24% of primary prevention patients and 46% of secondary prevention patients received lipid-lowering therapy [26]. Similarly, lipid-lowering therapy was prescribed in only ~29% of Chinese diabetic patients in a prospective cohort analysis of the Hong Kong Diabetes Registry [27].

Numerous factors likely contribute to the gap between treatment recommendations for CV risk management in diabetes and real-world clinical settings. For example, while LDL-C-targeted statin therapy is advocated for the pharmacological management of dyslipidemia in patients with diabetes [4], many U.S. physicians hold falsely elevated concerns about the perceived risk of hepatotoxicity that prevent statin prescription in patients with a clinical indication for their use [28]. Such concerns are not supported by current evidence from randomized trials and meta-analyses demonstrating the safety and tolerability of statin therapy, particularly in patients with diabetes [10-13,29], and recommendations to discontinue the routine monitoring of liver function tests in statin-treated patients [30].

### Reducing CV risk in diabetes: evidence from randomized controlled trials

Although suboptimal management of CV risk factors persists in U.S. adults with diabetes, the observed increases in the use of lipid-lowering agents and LDL-C goal attainment, particularly in the latter half of the past decade, may correspond to the publication of several large CV outcomes trials supporting the use of statins in this high-risk population. In 2003, a subgroup analysis of patients with diabetes enrolled in the Heart Protection Study (HPS) [10] reported that treatment with simvastatin reduced the relative risk of first major CV events by 22%. Reductions in relative risk of up to one-third were observed in diabetic patients without a diagnosis of CHD at study entry and/or whose pretreatment LDL-C level was <3.00 mmol/L (116 mg/dL) [10], suggesting that lipid-lowering therapy is beneficial for patients with diabetes even in the absence of overt CHD or elevated lipid levels. The 2004 publication of the Collaborative Atorvastatin Diabetes Study (CARDS) [11], which investigated the effectiveness of atorvastatin for primary prevention of CV events in patients with diabetes and baseline LDL-C levels ~3.03 mmol/L (117 mg/dL), reinforced this notion. Randomization to atorvastatin was associated with a 37% reduction in the relative risk of major CV events; the relative risk of stroke was reduced by 48% [11]. More recent subgroup analyses from other large statin trials, such as the Anglo-Scandinavian Cardiac Outcomes Trial-Lipid-Lowering Arm (ASCOT-LLA) [12] and the Treating to New Targets (TNT) study [13], have continued to provide evidence on the benefits of statin therapy in reducing CV complications in diabetic individuals.

Similarly, CV outcomes trials of antihypertensive agents demonstrating the clinical benefit of aggressive treatment to BP targets in patients with diabetes may also have contributed to the significant increase in the use of BP-lowering medications by this group in the past decade. In 1998, the U.K. Prospective Diabetes Study (UKPDS) compared tight control of BP to <150/85 mmHg versus less-tight control of BP to <180/105 mmHg in hypertensive diabetic patients [14]. The observed reductions in relative risk in patients allocated to tight BP control were 24% for diabetes-related end points (which included sudden death, myocardial infarction, angina, heart failure, and stroke), 32% for deaths related to diabetes (which included death due to myocardial infarction, sudden death, stroke, and peripheral vascular disease), and 44% for stroke [14]. A subgroup analysis of the diabetic cohort within the Hypertension Optimal Treatment (HOT) trial, where hypertensive patients were randomly assigned to a target diastolic BP, found that the risk of major CV events was halved in those randomized to ≤80 mmHg versus ≤90 mmHg [15]. However, while trials such as these have generally indicated that aggressive BP control is effective in reducing the risk of CV complications of diabetes, recent results have questioned the clinical benefit of lowering BP levels to within the normotensive range. The Action to Control Cardiovascular Risk in Diabetes (ACCORD) trial, where diabetic patients were targeted to a systolic BP <120 mmHg versus <140 mmHg, did not demonstrate a significant reduction in the relative risk of major CV events but did show a significant reduction in stroke risk [31]. Similarly, a subgroup analysis from the International Verapamil SR-Trandolapril Study (INVEST) found that, among diabetic patients with CHD, strict control of systolic BP (<130 mmHg) was not associated with improved CV outcomes in comparison with usual control (130 mmHg to <140 mmHg) [32]. Nevertheless, despite recent debate around intensive BP control, the finding from our analysis that nearly half of all U.S. adults with diabetes are not meeting the current recommendations around LDL-C or BP treatment goals is of concern, and may have serious implications for the future CVD burden in this population.

### Improving quality of care in diabetes

The frontline in the battle to improve therapeutic goal attainment within the context of diabetes is the primary-care setting; hence, quality-of-care initiatives must engage both patients and providers. National awareness campaigns, such as those by the National Diabetes Education Program, serve to increase patient understanding of CV risk factors and their control through self-management. Provider recognition programs and public reporting of performance measures may motivate healthcare professionals to improve levels of diabetes care [33]. Other provider-level incentive strategies designed to enhance patient outcomes include educational (e.g., continuing medical education credit), practical (e.g., technical assistance for quality improvement activities), managerial (e.g., increased autonomy), and financial (e.g., pay-for-performance) components, which have been used either alone or in combination by individual health plans, employers, and government purchasers with varying degrees of success [33]. The Physicians Quality Reporting Initiative, implemented by the Centers for Medicare and Medicaid Services, is a pay-for-participation program where providers who satisfactorily report on a set of quality measures earn an incentive payment, and is viewed by many as a framework for a nationalized pay-for-performance scheme. An example of how a government incentive program might improve evidence-based standards of care in diabetes can be found in the United Kingdom where, in 2004, the National Health Service introduced a pay-for-performance scheme to reward primary-care practices that achieve clinical targets for a number of chronic conditions. Substantial improvements in the attainment of BP (≤145/85 mmHg) and total cholesterol (≤5.05 mmol/L [195 mg/dL]) goals in patients with diabetes have been observed since the introduction of the scheme, with as many as ~80% of patients achieving these (albeit less-stringent) targets by 2007-2008 [34]. However, further evaluation is needed to assess the contribution of this scheme to improvements in care versus underlying positive trends in quality, as well as the potential for selective inclusion or exclusion of patients and achievement-threshold effects [34].

### Study limitations

The results of our current analysis should be interpreted with consideration of the following limitations. Information on NHANES participants' drug utilization and disease history is derived from self-reported data obtained through interviews and questionnaires, and as such may be subject to recall bias. Also, laboratory measurements of glucose and lipid levels were performed on a single specimen, and relied on participants self-reporting an appropriate period of fasting, the absence of which may confound prevalence estimates and goal attainment rates. Although current national guidelines for the assessment of elevated cholesterol levels do not stipulate repeat testing for lipid measurements [8], the diagnostic criteria for diabetes recommend that any positive test result for diabetes in asymptomatic individuals should be confirmed by a repeat test on a subsequent day to rule out laboratory error [4]. While confirmation of a diagnosis of diabetes is of vital importance in the clinical setting, the incremental benefit derived from sequential testing of plasma glucose levels within an epidemiological context is less clear. While encouraging, the positive trend in HDL-C goal attainment rates must be interpreted with caution due to potential bias arising from the various HDL-C assays employed in NHANES during this period, despite adjustments made to HDL-C values to account for this [18]. The low number of participants with valid data on their diabetic status, in addition to complete lipid and BP data, in NHANES 1999-2008 limited the ability to perform and interpret subgroup analyses of the data. The exclusion of institutionalized persons in NHANES, such as those residing in long-term care, may have contributed to the low number of diabetic participants identified for inclusion in this analysis. Finally, NHANES data are derived from separate cross-sectional surveys for each study cycle. Hence, while trends in prevalence estimates and goal attainment rates can be inferred from the data, conclusions as to causality of these temporal relationships are not possible.

## Conclusions

Among the U.S. diabetic population, the proportion of individuals taking cholesterol- and BP-lowering medication has increased significantly in the past decade. However, while there has been a significant improvement in LDL-C goal attainment rates over time, nearly one-half of all U.S. adults with diabetes are currently not achieving LDL-C or BP treatment goals recommended by the latest clinical practice guidelines. Moreover, only ~1 in 4 people with diabetes are achieving both the LDL-C and BP treatment targets simultaneously. These nationally representative trends suggest that continued efforts by healthcare professionals are required to achieve recommended treatment goals for CV risk factors and reduce the economic and social burden of CV complications associated with diabetes.

## Competing interests

A.K. and J.M. are employees of Pfizer Inc. with ownership of stock in Pfizer Inc.

## Authors' contributions

AK designed the study and wrote the manuscript. JM performed the statistical analyses and wrote the manuscript. Both authors read and approved the final manuscript.
